# Highly Strong, Tough, and Cryogenically Adaptive Hydrogel Ionic Conductors via Coordination Interactions

**DOI:** 10.34133/research.0298

**Published:** 2024-01-12

**Authors:** Zhuomin Wang, Siheng Wang, Lei Zhang, He Liu, Xu Xu

**Affiliations:** ^1^ Institute of Chemical Industry of Forestry Products, Key Laboratory of Biomass Energy and Material, Jiangsu Province; Key Laboratory of Chemical Engineering of Forest Products, National Forestry and Grassland Administration; National Engineering Research Center of Low-Carbon Processing and Utilization of Forest Biomass; Jiangsu Co-Innovation Center of Efficient Processing and Utilization of Forest Resources, Chinese Academy of Forestry, Nanjing 210042, China.; ^2^ College of Chemical Engineering, Jiangsu Co–Innovation Center of Efficient Processing and Utilization of Forest Resources, Nanjing Forestry University, Nanjing 210037, China.

## Abstract

Despite the promise of high flexibility and conformability of hydrogel ionic conductors, existing polymeric conductive hydrogels have long suffered from compromises in mechanical, electrical, and cryoadaptive properties due to monotonous functional improvement strategies, leading to lingering challenges. Here, we propose an all-in-one strategy for the preparation of poly(acrylic acid)/cellulose (PAA/Cel) hydrogel ionic conductors in a facile yet effective manner combining acrylic acid and salt-dissolved cellulose, in which abundant zinc ions simultaneously form strong coordination interactions with the two polymers, while free solute salts contribute to ionic conductivity and bind water molecules to prevent freezing. Therefore, the developed PAA/Cel hydrogel simultaneously achieved excellent mechanical, conductive, and cryogenically adaptive properties, with performances of 42.5 MPa for compressive strength, 1.6 MPa for tensile strength, 896.9% for stretchability, 9.2 MJ m^−3^ for toughness, 59.5 kJ m^−2^ for fracture energy, and 13.9 and 6.2 mS cm^−1^ for ionic conductivity at 25 and −70 °C, respectively. Enabled by these features, the resultant hydrogel ionic conductor is further demonstrated to be assembled as a self-powered electronic skin (e-skin) with high signal-to-noise ratio for use in monitoring movement and physiological signals regardless of cold temperatures, with hinting that could go beyond high-performance hydrogel ionic conductors.

## Introduction

The ever-increasing surge for high-performance ionic conductors with some unusual uses has been spurred by the growing demand for flexible and wearable electronics. Owing to the distinctive combination of conductivity, stretchability, and conformability, conductive polymer hydrogels have been regarded as a potential candidate for intelligent electronics, which have made it feasible for diverse modern technologies across different fields, including energy harvesting/storage devices, epidermal electronics, and wearable sensors [1–6]. However, conventional polymer hydrogels, due to poor interchain interactions, are inherently brittle and fragile and weakly resist cracking, making them prone to failure in practical use [7–9]. Moreover, most of the existing hydrogels are prone to freezing at low temperatures, which perceivably restricts their ability to function in a variety of challenging application scenarios [10–12]. Therefore, there is an urgent expectation—hydrogel ionic conductors integrating excellent mechanical, electrical, and cryoadaptive properties—for the ever-changing real world.

Existing developed tough hydrogel ionic conductors are usually prepared by several design approaches, such as increasing polymer crosslink density, constructing double network hydrogels, creating nanocomposite systems, and exploiting multiple bonding interactions [13–16]. Unfortunately, these manipulations often bring contradictory feedbacks, and a compromise emerges, that is, enhanced mechanical strength accompanied by degraded ionic conductivity, which seriously affects the targeted functionality of hydrogel ionic conductors [17,18]. Also, complicated operations and cumbersome procedures, as well as compatibility concerns, are inevitably involved [19]. For example, the construction of double network hydrogel ionic conductors tends to require elaborate steps or conditions [20]. Additionally, these production techniques only contribute to the mechanical enhancement of hydrogel ionic conductors in one way and have to undergo secondary modification to optimize the conductive and cryogenically adaptive performance, which greatly increases the production process [21,22]. Undeniably, despite the recent advancements in physically robust hydrogels, the direct development of highly mechanical, tough, and cryotolerant all-around hydrogel ionic conductors in a facile yet effective manner has faced lingering challenges.

**Fig. 1.**
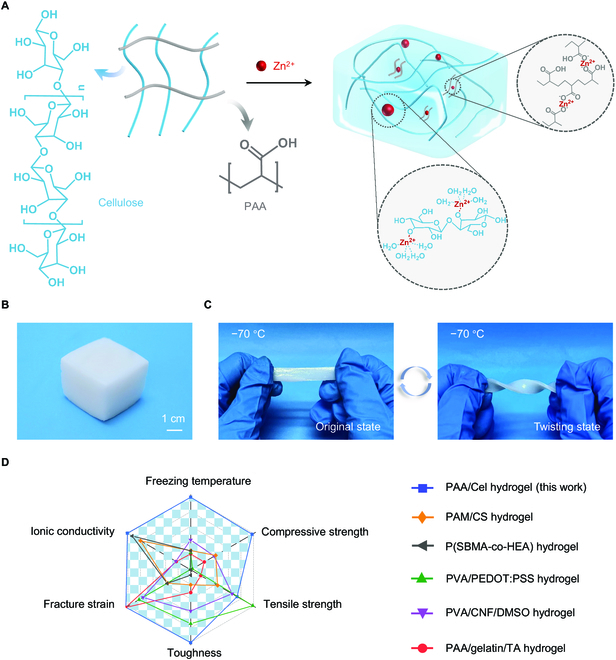
Fabrication of the PAA/Cel hydrogel. (A) Schematic of the developed microstructure from the PAA/Cel hydrogel. (B) Photograph of the PAA/Cel hydrogel. (C) Photographs of the PAA/Cel hydrogel with original and twisting state at −70 °C. (D) A comprehensive comparison between the PAA/Cel hydrogel from this work and previously reported robust counterparts in several key metrics including freezing temperature, compressive strength, tensile strength, toughness, fracture strain, and ionic conductivity.

Here, we report an all-in-one strategy to fabricate hydrogel ionic conductor with excellent mechanical, toughness, and freezing resistance to overcome these dilemmas through coordination interactions. The hydrogel ionic conductor consists of acrylic acid (AA) for a common monomer and cellulose for biomass resource in situ polymerization, in which cellulose is obtained by dissolving in a zinc chloride/aluminum chloride (ZnCl_2_/AlCl_3_) binary solvent system at ambient temperature (Fig. 1A). In particular, the oxygen-containing polar functional groups (i.e., hydroxyl groups) found in the repeating anhydroglucose units that make up the cellulose molecular chains are abundant in the hierarchical structure of cellulose, which have been demonstrated to be capable of producing stable coordination bond with multivalent metal units [23–28]. Therefore, the resultant poly(acrylic acid)/cellulose (PAA/Cel) hydrogel contains abundant concentrated ionic salts, where zinc ions easily form coordination with carboxyl groups from PAA and hydroxyl groups from cellulose, allowing the dramatic improvement in mechanical performance. In addition, the PAA/Cel hydrogel is rich in active ions, which allows the hydrogel to resist freezing failure caused by the aggregation of water molecules at low temperatures. On the other hand, the PAA/Cel hydrogel also features high ionic conductivity regardless of a wide temperature range. As a result, it can be found that the obtained PAA/Cel hydrogel is easily prepared into complex shapes, and a 5 cm × 5 cm × 4 cm hydrogel sample remains in its initial shape at ambient temperature for 3 days with negligent liquid leakage and holding three-dimensional (3D) porous architecture, demonstrating considerable environmental stability (Fig. 1B and Fig. S1). Encouragingly, the PAA/Cel hydrogel maintains high conformability even when subjected to −70 °C and can be repeatedly twisted without obvious cracking and shattering, revealing its reliable cryotolerant performance (Fig. 1C). Benefiting from the combination of strong, tough, and cryogenically adaptive properties of the PAA/Cel hydrogel, the developed hydrogel is more competitive than reported counterparts in several important metrics including freezing temperature, compressive strength, tensile strength, toughness, fracture strain, and ionic conductivity (Fig. 1D and Table S1) [22,29–32]. The presented all-in-one strategy has great potential and open up new avenues for the sample yet effective preparation of hydrogel ionic conductors, which could lead to significant applications in healthcare management, intelligent monitoring, and human–machine interface whether in daily life or in extremely cold environments.

## Results and Discussion

### Characterization of zinc-coordinated interactions

**Fig. 2.**
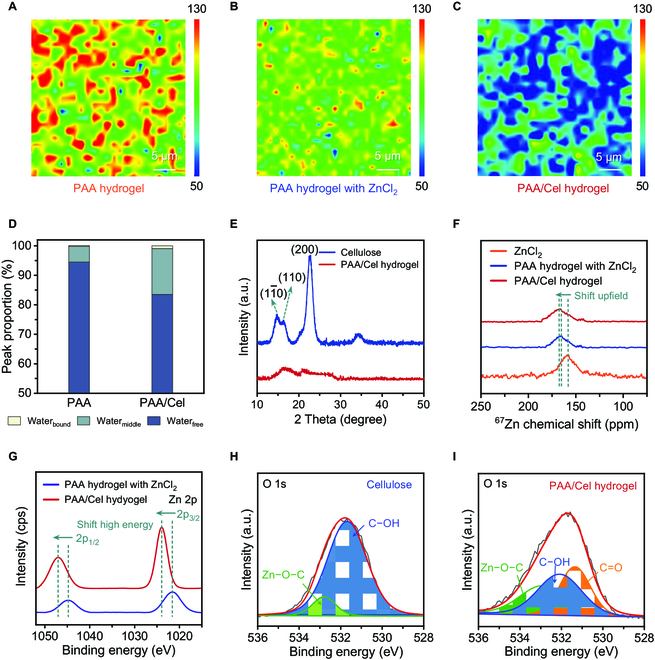
Structure analysis of the PAA/Cel hydrogel. (A to C) Two-dimensional (2D) Raman images of the PAA hydrogel (A), PAA hydrogel with ZnCl_2_ (B), and PAA/Cel hydrogel (C) were obtained from C=O (1600 to 1800 cm^−1^) stretching vibration. (D) LF NMR spectra of the PAA hydrogel and PAA/Cel hydrogel. (E) XRD patterns of the cellulose and PAA/Cel hydrogel at diffraction angle (2*θ*) range from 10° to 50°. (F) ^67^Zn NMR spectra of the ZnCl_2_ solution, PAA hydrogel with ZnCl_2_, and PAA/Cel hydrogel. (G) XPS spectra of the PAA hydrogel with ZnCl_2_ and PAA/Cel hydrogel. (H and I) O 1s XPS orbital spectrum for the Zn^2+^-dissolved cellulose (H) and the PAA/Cel hydrogel (I).

**Fig. 3.**
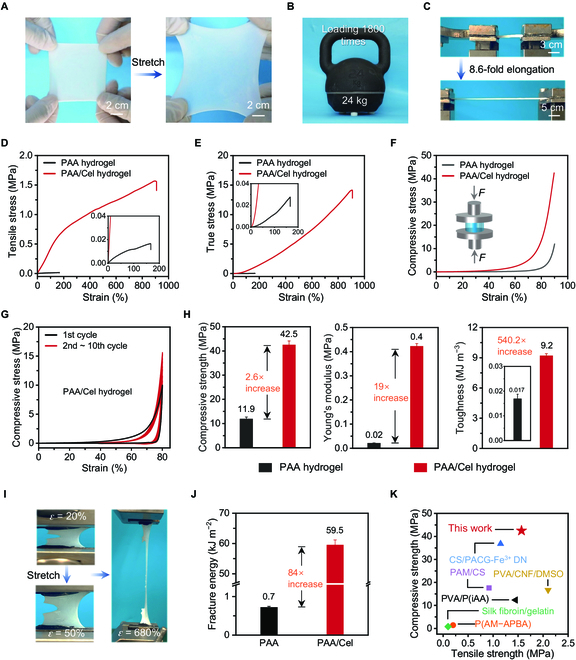
Mechanical properties of the PAA/Cel hydrogel. (A to C) Photographs of the PAA/Cel hydrogel pulled (A), compressed (B), and stretched (C) under external forces. (D) Tensile stress–strain curves of hydrogels. (E) True tensile stress–strain curves of hydrogels. (F) Compressive stress–strain curves of hydrogels. (G) Cyclic compressive stress–strain curves of the PAA/Cel hydrogel at a fixed strain of 80%. (H) A comparison of compressive strength, Young’s modulus, and toughness of hydrogels. (I) Photographs of the notched PAA/Cel hydrogel for the pure shear test. (J) A comparison of fracture energy for the PAA hydrogel and PAA/Cel hydrogel. (K) Tensile and compressive properties of the PAA/Cel hydrogel and other recently reported robust polymer hydrogels.

Figure 2A to C compares the 2D Raman mapping images of PAA hydrogel, PAA hydrogel with ZnCl_2_, and PAA/Cel hydrogel to observe the subtle micro-crosslinking structure significance, where the blue-green-colored area represents higher crosslink densities, while the red-yellow-colored area represents the opposite. Surprisingly, the PAA/Cel hydrogel exhibits higher crosslink densities compared to the PAA hydrogel, which is due to that cellulose with Zn^2+^ salts contributes a lot in increasing crosslink densities. As expected, the PAA hydrogel with ZnCl_2_ exhibits similar crosslink densities compared to the PAA hydrogel, which is caused by decreased crosslink sites due to the lack of cellulose. These impressive observations are in good agreement with the low-field nuclear magnetic resonance (LF NMR) data, that is, decreased free water as well as increased immediate water and bound water are seen in the PAA/Cel hydrogel compared to the PAA hydrogel, further demonstrating that cellulose with Zn^2+^ salts enables higher crosslink densities to limit the movement of water in the hydrogel (Fig. 2D and Fig. S2). Furthermore, Fig. 2E and Fig. 3 show the x-ray diffraction (XRD) patterns of the pristine cellulose and the dry PAA/Cel hydrogel sample. The three characteristic diffraction peaks of pristine cellulose located at 2*θ* = 14.7°, 16.2°, and 22.6° are attributed to the (1$\bar{1}$0), (110), and (200) planes, respectively [27,33]. The intensity and sharpness of these representative diffraction peaks significantly decrease for the dry PAA/Cel hydrogel sample, suggesting a degraded crystallinity of cellulose [34]. This is reasonably explained by the salt dissolution system destroying the original crystalline region of cellulose, as well as the formation of reconstructed coordination interactions and hydrogen bonds between cellulose and Zn^2+^, and between cellulose and PAA, respectively [35].

^67^Zn NMR spectroscopy was performed to learn more about molecular interactions in the PAA/Cel hydrogel (Fig. 2F). The PAA/Cel hydrogel (167.7 ppm) and the PAA hydrogel with Zn^2+^ (165.6 ppm) samples show an upfield chemical shift in comparison to the ZnCl_2_ aqueous (158.1 ppm) according to the ^67^Zn NMR spectra [36,37]. This suggests that hydrogels containing Zn^2+^ have a representative deshielding effect. The solvation structure variation in hydrogel system is responsible for the trigger of Zn^2+^ through the development of Zn-PAA and Zn-cellulose coordination interactions [36,38]. In this regard, we further conducted the x-ray photoelectron spectroscopy (XPS) to analyze the Zn bonding in the PAA/Cel hydrogel (Fig. 2G). The characteristic satellite peaks of Zn 2p XPS appear simultaneously in the two samples, confirming the presence of Zn^2+^ in Zn-coordinated hydrogels. In particular, these two characteristic satellite peaks in the Zn 2p XPS of the PAA/Cel hydrogel exhibit a significant deviation that shifts higher energy compared with that of the PAA hydrogel with the ZnCl_2_ sample, that is, Zn 2p_1/2_ peaks from 1044.8 eV to 1047.0 eV, as well as Zn 2p_3/2_ peaks from 1021.5 eV to 1024.0 eV, which also supports the formation of Zn-PAA and Zn-cellulose coordination interactions [39,40]. Furthermore, we verified the formation of Zn−O−C bonds in the PAA/Cel hydrogel system by O 1s XPS orbital spectrum (Fig. 2H and I). It can be found that in Zn^2+^-dissolved cellulose, there are two deconvoluted peaks, representing the C−OH peak located at 531.7 eV and the Zn−O−C peak located at 532.8 eV, indicating that Zn^2+^ and cellulose can form Zn−O−C bonds (Fig. 2H) [41,42]. In contrast, for the PAA/Cel hydrogel, we observed a new peak at 531.3 eV belonging to C=O groups, which delivers a decreased C−OH ratio and an increased Zn−O−C ratio (obtained by calculating the integrated area of the corresponding deconvoluted peak curve), revealing that Zn^2+^ is capable of forming Zn−O−C bonds with both PAA and cellulose (Fig. 2I and Fig. S4). These XPS results indicate a change in the orbital electron density of zinc, which is caused by the higher binding energy between Zn^2+^ and carboxyl and hydroxyl groups. The mapping techniques and spectroscopic analysis results jointly evidence that Zn^2+^ in the hydrogel system is readily coordinated and O from PAA and cellulose to form Zn−O−C bonds.

### Mechanical properties of zinc-coordinated hydrogels

Benefiting from the favorable zinc coordination interaction, the obtained PAA/Cel hydrogel undergoes significant deformation under external force without visible cracks and fractures, and still shows non-obvious breaks even during loading of 1,800 times its own weight, indicating excellent toughness and robustness (Fig. 3A and B). It is surprising that the hydrogel can be stretched up to 8.6-fold elongation, exhibiting fascinating stretchability (Fig. 3C).

The PAA/Cel hydrogel exhibits great advantages in terms of both tensile stress and fracture strain in comparison to the PAA hydrogel; specifically, its tensile strength is 1.6 MPa at a fracture strain of 896.9%, while the PAA hydrogel is 0.02 MPa and 166.1%, indicating improvements of 79.0 and 4.4 times, respectively (Fig. 3D). We attribute this to the strong zinc-coordinated interactions formed between Zn^2+^ and PAA/Cel. The true stress and associated strain curves of the hydrogel are achieved because the PAA/Cel hydrogel exhibits good stretchability, leading to a smaller cross-sectional area during deformation [43]. It can be found that the true stress of the PAA/Cel hydrogel reaches 14.1 MPa, indicating its excellent mechanical strength (Fig. 3E). Simultaneously, the highest compressive strength of the PAA/Cel and PAA hydrogel is 42.5 and 11.9 MPa, respectively. This is a 2.6-fold increase at a compressive strain of 90%, while a 7.0-fold increase is exhibited at a compressive strain of 80%, further evidencing the superior mechanical strength for the PAA/Cel hydrogel (Fig. 3F and Fig. S5). Furthermore, cyclic compressive stress–strain curves were obtained from the PAA/Cel hydrogel at a fixed compressive strain of 80% (Fig. 3G). Interestingly, it can be observed that after 10 cyclic compression tests, the PAA/Cel hydrogel exhibits an increased compressive stress at the ultimate strain, implying that the durable mechanical stability is achieved by zinc-coordinated interactions facilitated to resist deformation (Figs. S6 and S7). In addition, the PAA/Cel hydrogel exhibits a tensile modulus increase of 19.0 times (0.4 MPa versus 0.02 MPa) and a fracture toughness increase of 540.2 times (9.2 MJ m^−3^ versus 0.017 MJ m^−3^) compared to the PAA hydrogel (Fig. 3H and Fig. S8). These results show that zinc-coordinated interactions can lead to a significant increase in hydrogel strength and toughness. It is noteworthy to notice that the PAA/Cel hydrogel demonstrates superior mechanical characteristics in tension and compression compared with the PAA hydrogel with ZnCl_2_, hence validating the beneficial synergy between cellulose and zinc-coordinated interactions (Fig. S9). Additionally, we found that the PAA/Cel hydrogel still maintains considerable mechanical properties even after being soaked in water and saline for up to 20 h (Figs. S10 and S11).

The single-notch tensile method was used to stretch the notched hydrogels and record the critical strain at which crack propagation begins in pure shear tests, which were conducted to assess the fracture resistance of the PAA/Cel hydrogel [44,45]. The notched PAA/Cel hydrogel was found to be stretched to a strain of 680%, which is roughly equal to the maximal strain of the unnotched hydrogel (Fig. 3I). This finding suggests that the hydrogel is nearly impervious to cracks. Indeed, the fracture energy (*Γ*) of the PAA/Cel hydrogel reaches 59.5 kJ m^−2^, which is significantly higher than that of 0.7 kJ m^−2^ for the PAA hydrogel, and 38.4 kJ m^−2^ for the PAA hydrogel with ZnCl_2_, further revealing its excellent fracture resistance (Fig. 3J and Fig. S12). As a result, we compare the prepared hydrogels with recently reported robust polymer hydrogels, that is, on account of the strong zinc-coordinated interactions in the hydrogel systems, the obtained PAA/Cel hydrogel delivers remarkable advantages in both compressive strength and tensile strength (Fig. 3K and Table S2) [22,32,46–49].

### Cryogenically adaptive properties of zinc-coordinated hydrogels

**Fig. 4.**
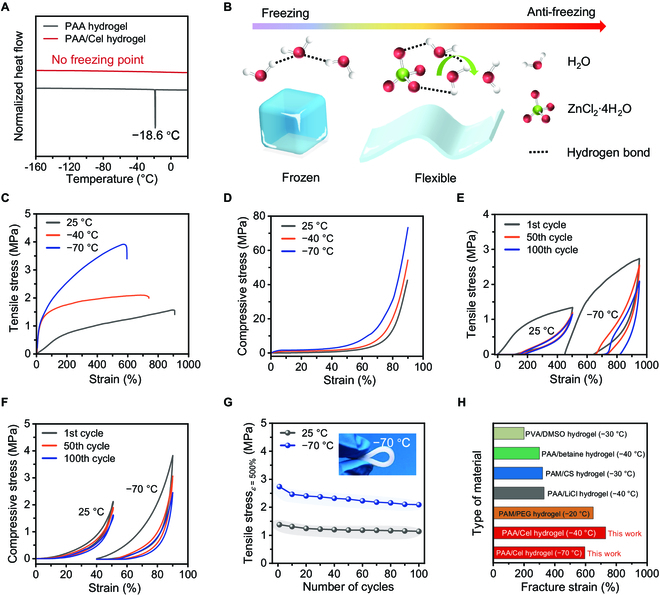
Cryogenically adaptive properties of the PAA/Cel hydrogel. (A) DSC curves of the PAA hydrogel and PAA/Cel hydrogel. (B) An intuitive illustration of freezing resistance for the PAA/Cel hydrogel. (C and D) Tensile (C) and compressive (D) stress–strain curves of the PAA/Cel hydrogel at 25, −40, and −70 °C, respectively. (E) Cyclic tensile stress–strain curves of the PAA/Cel hydrogel at a fixed strain of 500% at 25 and −70 °C. (F) Cyclic compressive stress–strain curves of the PAA/Cel hydrogel at a fixed strain of 50% at 25 and −70 °C. (G) Corresponding stress of the PAA/Cel hydrogel at maximum tensile strain of 500% at 25 and −70 °C. (H) Comparison of the low-temperature fracture strain for the PAA/Cel hydrogel and recent anti-freezing hydrogels.

Benefiting from the presence of substantive electrolytic salt in the PAA/Cel hydrogel, it can be seen that no exothermic peak appears in the hydrogel even at −160 °C because of the coagulation of water in the system by the differential scanning calorimeter (DSC) tests, indicating that the PAA/Cel hydrogel has excellent freezing resistance (Fig. 4A). In contrast, the PAA hydrogel has a freezing point of −18.6 °C, which represents that the water molecules inside the hydrogel networks are susceptible to freezing. Figure 4B and Fig. S13 provide an intuitive illustration of freezing resistance; for the hydrogel containing fewer free ions, such as the PAA hydrogel, when the temperature drops below the freezing point, the water molecules inside the hydrogel are poised to rapidly aggregate to form ice because of hydrogen bonds between water molecules, thereby resulting in the formation of a frozen hydrogel. Because the ions can be tightly combined with water molecules to weaken the hydrogen bonds between water molecules, the PAA/Cel hydrogel with more free ions (such as ZnCl_2_) is able to limit the aggregation of large amounts of water molecules and eventually enable a flexible hydrogel even at lower temperatures [50].

To assess the anti-freezing capabilities of zinc-coordinated hydrogels, a comprehensive investigation was conducted into the mechanical properties of the PAA/Cel hydrogel at low temperatures. It is observed that the PAA hydrogel is frozen at −20 °C, unable to resist bending and fracture; in striking contrast, the PAA/Cel hydrogel can withstand conspicuous deformation even at −70 °C without obvious breaks (Fig. S14). According to quantitative analysis of the tensile stress–strain curves displayed in Fig. 4C and Fig. S15, the PAA/Cel hydrogel shows a decrease in fracture strain and an increase in maximum tensile stress when the temperature drops, which is a common mechanical behavior in polymers due to the low temperature limiting the chain movement. Notably, the PAA/Cel hydrogel exhibits softness and flexibility similar to that at 25 °C even at temperatures as low as −70 °C, with tensile strength and fracture strain of 3.9 MPa and 595.2%, respectively. As expected, the PAA/Cel hydrogel presents compressibility even at −70 °C with a compressive strength of 73.4 MPa, likewise revealing its considerable mechanical properties at low temperatures (Fig. 4D and Fig. S16). In addition, owing to the strong binding between free ions and water molecules in the PAA/Cel hydrogel network, the hydrogel also retains it relatively stable mechanical properties after being stored at 35 °C for 6 h (Fig. S17).

Furthermore, the durability of the PAA/Cel hydrogel at low temperature was conducted by cyclic tension and compression (Fig. 4E and F and Fig. S18). We compare the multiple stretch behavior of the PAA/Cel hydrogel at 25 and −70 °C at a fixed strain of 500%. It can be observed that, compared with 25 °C, the hydrogel exhibits similar mechanical behavior at −70 °C, that is, the stretching curve at the first cycle shows a significant energy hysteresis feature, which is due to the presence of noncovalent hydrogen bonds in the hydrogel that dissipate a significant amount of energy when deforming, thus maintaining the integrity of the hydrogel; almost overlapping closed curves are delivered after the 50th and 100th cycle of tension, indicating excellent cyclic stretchability even at low temperature (Fig. 4E). This can also be demonstrated by the cyclic compression behavior of the PAA/Cel hydrogel at −70 °C at a fixed strain of 50% through the observation that displays a small hysteresis energy after 100th consecutive compressions (Fig. 4F). More intuitively, we record the stress of the PAA/Cel hydrogel under varying continuous cycles at a fixed maximum tensile strain at 25 and −70 °C, and high stress retention is simultaneously delivered at different temperatures, suggestive of excellent mechanical durability even at low temperatures (Fig. 4G). Despite after 100th cycles in tension, the hydrogel remains flexible and bendable (inset in Fig. 4G). These are also in good agreement with the loop compression results (Fig. S19). With a good combination of freeze-tolerant and stretchable features, the resultant PAA/Cel hydrogel presents a significant advantage in low-temperature fracture strain compared to recent anti-freezing hydrogels (Fig. 4H and Table S3) [17,32,43,51,52].

### Sensing performance of zinc-coordinated hydrogels

**Fig. 5.**
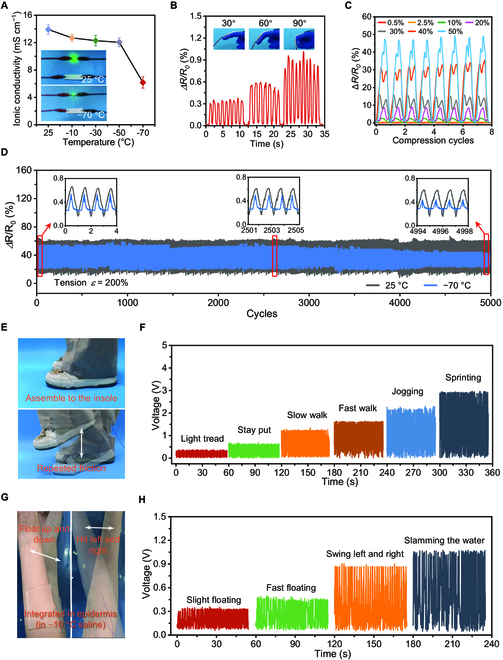
Sensing performance of the PAA/Cel hydrogel-based self-powered e-skins and real-world applications. (A) Ionic conductivity of the PAA/Cel hydrogel at varying temperatures. (B) Resistance changes of the PAA/Cel hydrogel ionic conductor assembled into e-skins under different bending angles of fingers. (C) Resistance changes of the PAA/Cel hydrogel ionic conductor assembled into e-skins during cycling compression at various strains (0.5%, 2.5%, 10%, 20%, 30%, 40%, and 50%). (D) Real-time responses by stretching the PAA/Cel hydrogel ionic conductor to 200% strain for 5000 cycles at 25 and −70 °C. (E and F) Photographs of the PAA/Cel hydrogel-based self-powered e-skins assembled to the insole (E) and the corresponding voltage output signal during varying motion including light tread, stay put, slow walk, fast walk, jogging, and sprinting (F). (G and H) Photographs of the PAA/Cel hydrogel-based self-powered e-skins integrated into the epidermal tissue (G) and the corresponding voltage output signal during varying motions including slight floating, fast floating, swing left and right, and slamming at −10 °C saline (H).

Owing to the good merger of abundant dissociable salts and low-temperature adaptability for the PAA/Cel hydrogel, the ionic conductivity reaches 13.9 mS cm^−1^ at 25 °C and 6.2 mS cm^−1^ at −70 °C, which indicates an excellent conductive property regardless of the varying temperature (Fig. 5A and Fig. S20). Notably, the ionic conductivity of the PAA/Cel hydrogel is significantly higher at 25 and −70 °C than that of the PAA hydrogel, and marginally lower than that of the PAA hydrogel containing ZnCl_2_, which can be explained by the partial formation of Zn−O−C bonds by free zinc ions (Figs. S21 and S22). It makes sense that even after squeezing, stretching, twisting, bending, and kinking the hydrogel at −70 °C to function as an ionic conductor in a closed circuit, a brilliant light-emitting diode (LED) light is still produced, revealing the potential sensing properties despite working at low temperatures (inset in Fig. 5A and Figs. S23 and S24).

In order to track actual human motions, we additionally put the hydrogel on a finger joint. It is discovered that the resistance change rate signal fluctuates continuously and repeatedly with the same amplitude during the bending cycle at a fixed bending angle, and that the resistance change rate increases with increasing bending angle (Fig. 5B). When gradually increasing the compressive strain of the PAA/Cel hydrogel from 0% to 75%, the value of the resistance change rate also increases stepwise (Fig. S25). As the resistance change rate rises from 4% to 48% over the course of eight compressive loading–unloading cycles, the PAA/Cel hydrogel also demonstrates a stable and consistent response to a wide range of compressive strains, from a small 0.5% to a large 50% (Fig. 5C and Fig. S26). To determine the durability of the PAA/Cel hydrogel as a flexible sensor over a broad operating temperature range, further long-term fatigue experiments were carried out (Fig. 5D and Fig. S27). For approximately 5,000 tension cycles at 200% strain and 1,000 compression cycles at 50% strain at both 25 and −70 °C, negligible degradation and the same amplitude of electrical signals were provided. Notably, it can be observed that the amplitude signal of the PAA/Cel hydrogel ionic conductor at −70 °C is smaller than the corresponding one at 25 °C, which is mainly due to the lower ionic conductivity of the PAA/Cel hydrogel ionic conductor at −70 °C. At −70 °C, when cyclic sensing exceeds 3,000 times, a slight decrease in amplitude is captured, which can be explained by the ultra-low temperature limiting the movement of polymer molecular chains in the hydrogel, thus yielding a certain degree of hysteresis during continuous loading and unloading at a tensile strain of 200% [53,54]. These highlight how the hydrogel works as a sensor in real-world applications because of its robust structure and consistent stability regardless of operating temperature.

Combining the strong mechanical capabilities, high ionic conductivity, and excellent low temperature resistance of the PAA/Cel hydrogel, it can be used as a conductive layer and polydimethylsiloxane (PDMS) elastomer as a friction layer to be integrated into a triboelectric nanogenerator (TENG) (Fig. S28 and Table S4) [55]. This integrated TENG shows excellent contact between the hydrogel and the PDMS elastomer without separation, as well as can harvest energy during physical activity and output it as a voltage signal for responding to the movement of the human body (Fig. S29). This eliminates the need for intricate procedures and avoids the need for an external energy source, and it may be built as a self-powered electronic skin (e-skin) for advanced sensing. First, we investigated the electrical output capability of the assembled TENG; increasing short-circuit current and open-circuit voltage with steady oscillations demonstrates good frequency responsiveness (Fig. S30). As a proof of concept, we integrated subsequently the designed TENG based on the PAA/Cel hydrogel as a self-powered e-skin for practical uses (Fig. 5E to H). When this e-skin is assembled into the insole during repeated rubbing, it exhibits a stepwise increasing voltage amplitude signal with a high signal-to-noise ratio as the stride frequency from slow for light tread to fast for sprinting, indicating an excellent sensory responsiveness (Fig. 5E and F). To further investigate the low-temperature application performance of the proposed e-skin, we integrated it as an epidermal electron to collect the motion signals of the arm in −10 °C saline (Fig. 5G). It can be observed that as the activity changes from light floating to slamming the water surface, an increasing response amplitude is delivered on the voltage with steady repetition, further revealing the outstanding sensing performance even at low temperature (Fig. 5H). Together, the self-powered e-skin developed based on the PAA/Cel hydrogel presents boosting potential as epidermal electronics for monitoring human movement and physiological signals despite the low operating temperature.

## Conclusion

In summary, we report an all-in-one strategy to develop hydrogel ionic conductors integrating excellent mechanical, electrical, and low-temperature adaptability through coordination interactions formed between zinc ions and PAA/Cel to address the lingering challenges in conducting hydrogels. Notably, this fabricated strategy is facile yet effective, that is, a variety of performance requirements can be realized by only one-step in situ polymerization of salt-dissolved cellulose and AA without additional operations or procedures. Enabled by a unique set of advantages, the resulting PAA/Cel hydrogel presents excellent mechanical properties in tension (for example, tensile strength of 1.6 MPa, strain levels of 896.9%, work of fracture of 9.2 MJ m^−3^, and fracture energy of 59.5 kJ m^−2^) and compression (an ultimate stress of 42.5 MPa) and conductive and cryoadaptive properties (ionic conductivity of 13.9 mS cm^−1^ at 25 °C and 6.2 mS cm^−1^ at −70 °C). As a proof of concept, the PAA/Cel hydrogel ionic conductor is used in flexible wearable self-powered e-skins for sensing body motion and physiological signals with verifiable output responses over a wide temperature, displaying great potential for real-world applications. The strategy displayed here contributes to the design of high-performance hydrogel ionic conductors and advances beyond other important areas.

## Materials and Methods

The detailed experimental procedures are presented in the Supplementary Materials.

## Data Availability

The data relevant to this study are available from the corresponding author upon reasonable request.

## Supplementary Materials

Supplementary 1 Materials and Methods Figs. S1 to S30 Tables S1 to S4 References [56–58]
