# Supplementary material for: Highly Strong, Tough, and Cryogenically Adaptive Hydrogel Ionic Conductors via Coordination Interactions
Source: Research (Wash D C). 2024 Jan 12;7:0298. doi: 10.34133/research.0298 (PMC10786319; doi:10.34133/research.0298)
Supplement: Supplementary 1 — Materials and Methods Figs. S1 to S30 Tables S1 to S4 References [56–58] [file research.0298.f1.docx]

**Supporting information**

**Highly strong, tough and** **cryogenically adaptive hydrogel ionic conductors via coordination interactions**

**Zhuomin Wang^1,2†^, Siheng Wang^1†^, Lei Zhang^1^, He Liu^1*^, Xu Xu^2*^**

^1^Institute of Chemical Industry of Forestry Products, Key Laboratory of Biomass Energy and Material, Jiangsu Province; Key Laboratory of Chemical Engineering of Forest Products, National Forestry and Grassland Administration; National Engineering Research Center of Low-Carbon Processing and Utilization of Forest Biomass; Jiangsu Co-Innovation Center of Efficient Processing and Utilization of Forest Resources, Chinese Academy of Forestry, Nanjing 210042, China.

^2^College of Chemical Engineering, Jiangsu Co–Innovation Center of Efficient Processing and Utilization of Forest Resources, Nanjing Forestry University, Nanjing 210037, China.

**^†^**These authors contributed equally to this work.

*****Corresponding authors: [liuhe.caf@gmail.com](mailto:liuhe.caf@gmail.com) (He Liu); [xuxu200121@njfu.edu.cn](mailto:xuxu200121@njfu.edu.cn) (Xu Xu).

**Materials and Methods**

**Materials and chemicals**

Poplar pulp Poplar pulp used to come from Qingdao Ruilibo International Trade Co., Ltd. (Qingdao, China) Zinc chloride (ZnCl_2_, AR, ≥98%) and aluminum chloride hexahydrate (AlCl_3_·6H_2_O, AR, 98.0~100%) were of analytical grade and purchased provided by Nanjing Chemical Reagent Co., Ltd. (Nanjing, China).Acrylic acid (AA, AR, ≥98%), N, N'-methylenebis(acrylamide) (MBAA, 99%) and ammonium persulfate (APS, AR, ≥98%) were purchased from Shanghai Aladdin Biochemical Technology Co., Ltd. (Shanghai, China) and without further purification.

**Preparation of** **salt-dissolved cellulose**

According to previously reported work, anhydrous aluminum chloride, anhydrous zinc chloride and water have a molar ratio of 0.1: 0.9: 4 for salt-soluble cellulose. (31) Briefly, poplar pulp (1 g) was added to the above mixed salt solution (99 g) with continuous vigorous stirring at ambient temperature until a transparent salt-dissolved cellulose solution was obtained without any residue was formed eventually.

**Preparation of poly(acrylic acid)/cellulose (PAA/Cel) hydrogel**

Firstly, AA (2.5 g) was added to the salt-dissolved cellulose solution (5 g) and stirred continuously until evenly solution was obtained. Subsequently, MBAA (0.01 g) and APS (0.0625 g) were added to the homogeneous solution for stirring uniformly at ambient temperature to obtain the prepolymer solution. At last, the prepolymer solution was poured slowly into a planar or columnar mold and then thermally polymerized at 60 °C for 2 h to prepare the PAA/Cel hydrogel. As a comparison, the same preparation method was used to obtain the pure PAA hydrogel and PAA hydrogel with ZnCl_2_.

**Characterization**

The X-ray diffraction (XRD) spectra of cellulose, freeze-dried salt-dissolved cellulose, and freeze-dried PAA/Cel hydrogel were recorded with an X-ray diffractometer (Siemens D5000, Germany) at a scanning speed of 3° min^−1^ in the diffraction angle (2*θ*) range from 10° to 50°. Raman imaging microscopes (Thermo Scientific DXR2xi, USA) was used to test at an excitation laser wavelength of 532 nm to obtained the Raman spectra and spatial Raman profiles of hydrogels. Fourier transform infrared (FTIR) spectra of freeze-dried PAA hydrogel, PAA hydrogel with ZnCl_2_ and PAA/Cel hydrogel samples were recorded in a wavenumber range from 4000 to 400 cm^−1^ using a Fourier transform infrared spectrometer (Nicolet iS50, Thermo Fisher Scientific, USA). Low-field NMR (LF NMR) spectroscopy was used to test the distribution of free, middle and bound water within hydrogels (Suzhou Niumag Corporation, China). By means of MAS probes, all solid-state NMR experiments were tested by a 600 MHz spectrometer (AVANCE NEO, Bruker, Germany). Separate ^67^Zn MAS NMR spectra were obtained for cellulose, salt-dissolved cellulose and PAA/Cel hydrogel. X-ray photoelectron spectroscopy (XPS) was conducted to analyze the elemental binding energy and the internal chemical composition of hydrogels. The pore structure of the surface of hydrogels stained with a fluorescent dye solution can be tested using confocal laser scanning microscopy (CLAM) (LSM710, Zeiss, Germany). SEM images of the prepared PAA/Cel hydrogel was obtained using an environmental scanning electron microscope (SEM) (JSM-7600Fs, Hitachi, Japan).

**Tensile and compressive tests**

Tensile and compressive measurements of cellulose hydrogels were performed using a universal tester (UTM6503, Shenzhen SUNS Tester Co., Ltd., China) equipped with a 5000-N transducer for compression and a 100-N transducer for tension. Tensile tests were performed on rectangular hydrogel samples measuring approximately 2 mm × 25 mm in size using calipers. The tensile stress-strain curve was tested under continuous tensile at a speed of 10 mm min^−1^. The hydrogel sample for the compression test was required to be a cylinder with a diameter of 15 mm and a height of 10 mm, and the compression velocity was 2 mm min^−1^. Additionally, cyclic tensile and compressive tests were carried out by subjecting hydrogels to a continuous loading-unloading process under the same deformation. Three identical hydrogel samples were tested for each set of conditions to record the average.

**Single-edge notch tension tests**

A unilateral notched tensile test was performed using two different samples, unnotched and notched, to measure the *Γ* of the fracture energy. For the notched samples, used a sharp scalpel to make a flat notch on one side of the rectangular sample. The distance between the two tensile grips was kept 7.2 mm and the tensile speed was fixed at 10 mm min^−1^. The fracture energy (*Γ*) was calculated as follows:

$\Gamma=HW(\varepsilon_{\text{c}})$ (1)

where *ε*_c_ represents the fracture deformation ratio of notched sample, W represents the strain energy calculated by integration of the stress versus strain of unnotched specimens with the same dimensions stretched to the *ε*_c_ strain, H represents the gauge length.

**Ionic conductivity**

The electrochemical impedance spectra (EIS) of each hydrogel were obtained using an Electrochemical Impedance Spectroscopy (CORRTEST, CS310H, China) at a voltage of 100 mV and a frequency range of 10^5^ to10^−1^ Hz. The following formula was used to calculated the ionic conductivity of hydrogels:

$\sigma_{c}=\frac{L}{RA}$ (2)

where *L* represents the distance between two probes, *R* represents the resistance of the hydrogel, and *A* represents the cross-sectional area of the hydrogel.

**Assembly of triboelectric nanogenerators (TENGs)**

The fabricated PAA/Cel hydrogel was first dialyzed to neutrality, and subsequently the dialyzed PAA/Cel hydrogel was used as the adhesive substrate and silicone rubber (polydimethylsiloxane, PDMS) as the friction negative material to form a triboelectric nanogenerator (TENG). A signal readout line was combined with the hydrogel layer to facilitate the connection of the hydrogel electrodes to external circuits. A homogeneous mixture of commercially available resins consisting of parts A and B of 1: 10 w/w Sylgard184 was dropped onto a cured hydrogel layer, and the PDMS elastomer layer was obtained by curing at 80 °C for 3 h. The nanofriction layers used in this study were all 3 cm × 3 cm × 2 cm in size.


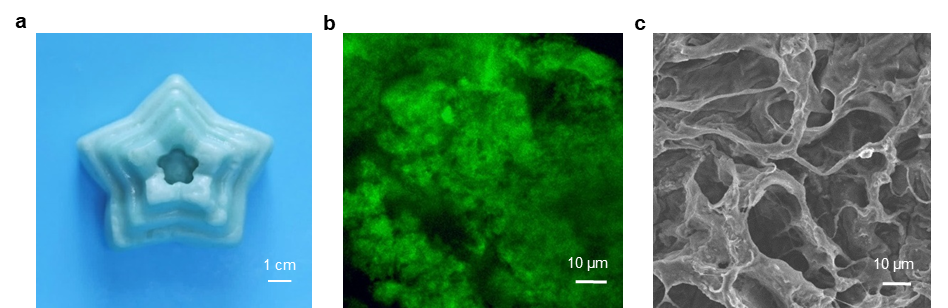


**Figure. S1 a** Photograph of the PAA/Cel hydrogel fabricated into complex shapes. **b** CLSM image of the PAA/Cel hydrogel. **c** SEM image of the PAA/Cel hydrogel.


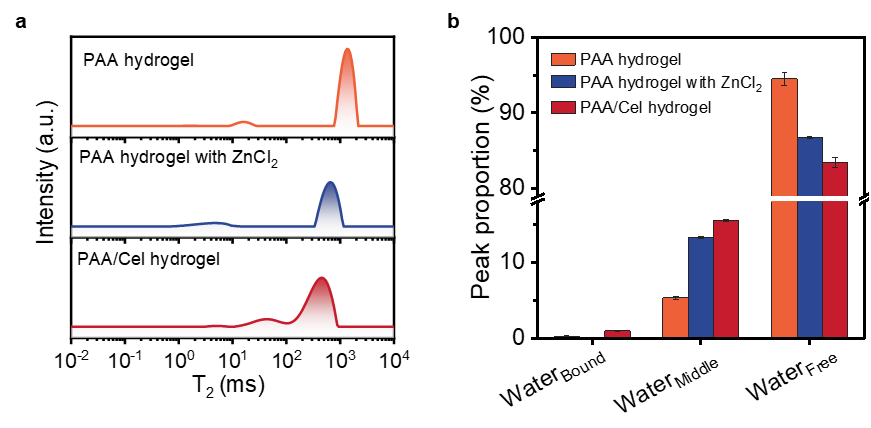


**Figure. S2 a, b** LF NMR spectra of hydrogels (**a**) and corresponding distributions of three types of water (**b**).

**Figure. S3** XRD patterns of the cellulose and salt-dissolved cellulose at diffraction angle (2*θ*) range from 10° to 50°.

**Figure. S4** O 1s XPS orbital spectrum for the PAA hydrogel with ZnCl_2_.


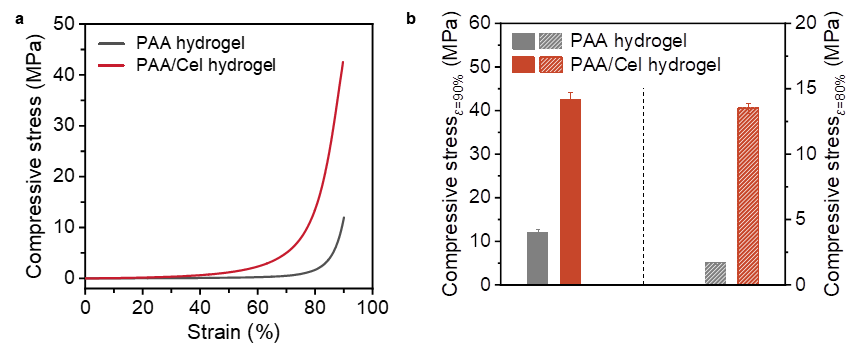


**Figure. S5** **a** Compressive stress-strain curves of the PAA hydrogel and PAA/Cel hydrogel. **b** Compressive stress of the PAA hydrogel and PAA/Cel hydrogel at strains of 90% and 80%.

A sharp contrast in compressive properties is presented in Fig. S5a. At the same ultimate compressive strain of 90%, the PAA/Cel hydrogel exhibits more excellent compressible properties compared to PAA hydrogel. Typically, the PAA/Cel hydrogel shows a stress of 42.5 MPa, while the PAA hydrogel corresponds to as low as 11.9 MPa, indicating a 2.6-fold increase due to the formation of coordinated covalent networks. As expected, a similar mechanical behavior was observed at a compressive strain of 80%, with the PAA/Cel hydrogel still has 8.0 times higher than that of the PAA hydrogel, delivering 13.6 MPa compared to 1.7 MPa, respectively (Fig. S5b).

**Figure. S6** Cyclic compressive stress-strain curves of the PAA hydrogel at a fixed strain of 80%.

**Figure. S7** Single-cycle compressive stress-strain curves of the PAA/Cel hydrogel at strain of 15%, 30%, 50%, 60%, 70%, and 80%.


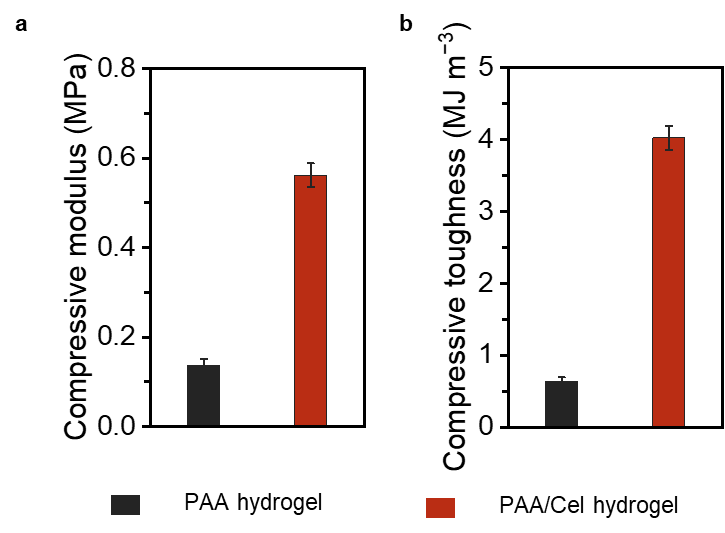


**Figure. S8 a, b** Compressive modulus (**a**) and toughness (**b**) of the PAA hydrogel and PAA/Cel hydrogel.


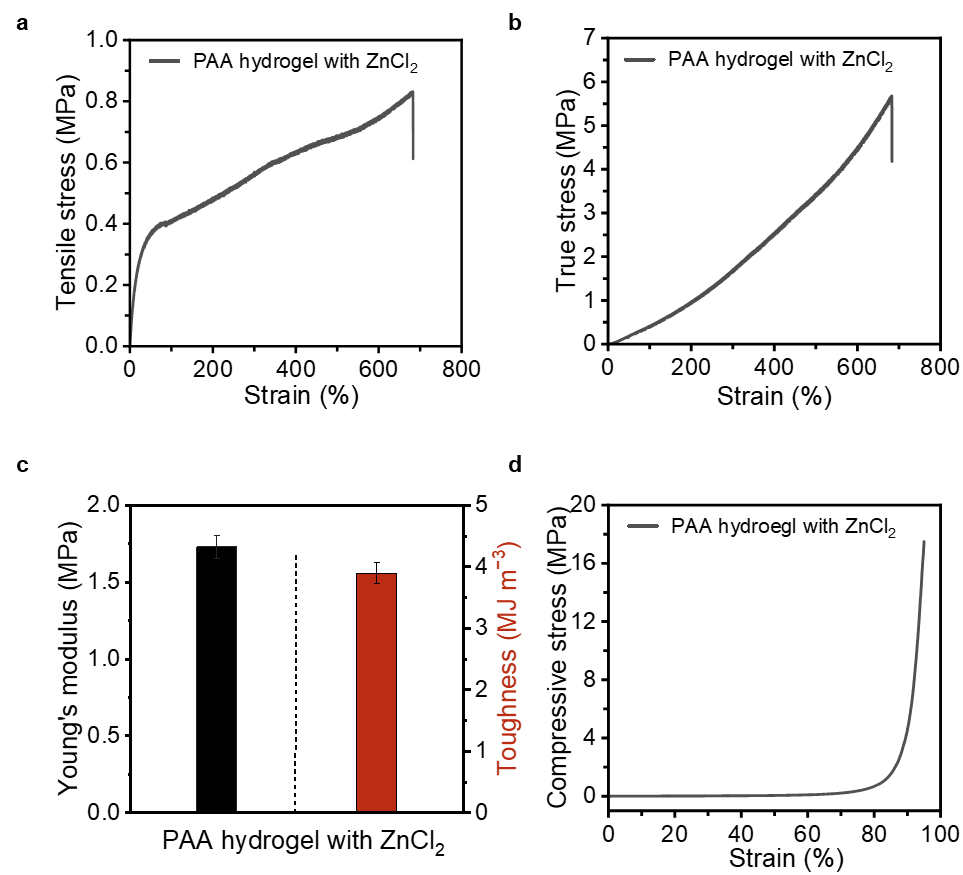


**Figure. S9 a, b** Tensile stress- (**a**) and true stress-strain (**b**) curves of the PAA hydrogel with ZnCl_2_. **c** A comparison of Young’s modulus and toughness for the PAA hydrogel with ZnCl_2_. **d** Compressive stress-strain curves of the PAA hydrogel with ZnCl_2_.


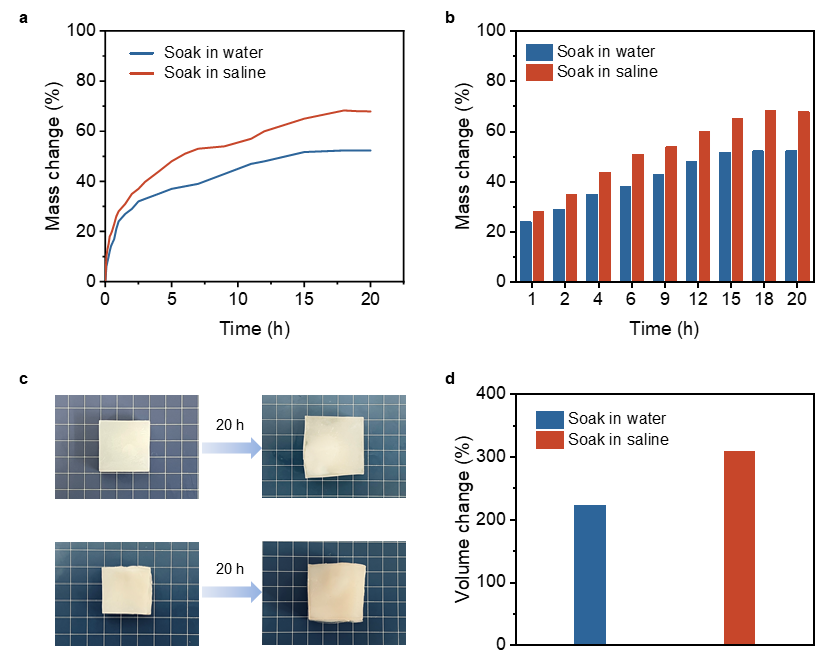


**Figure. S10 Swelling behavior of the PAA/Cel hydrogel in water and saline. a** Mass change versus time curves of the PAA/Cel hydrogel in water and saline. **b** Mass change trend of the PAA/Cel hydrogel in water and saline. **c** Photographs of the PAA/Cel hydrogel before and after soaking in water and saline for 20 h. **d** Volume change of the PAA/Cel hydrogel after stabilization in water and saline.

Fig. S10a shows the mass change-soaking time curves of the PAA/Cel hydrogel. It can be found that the hydrogel swell to a certain extent in water and saline, and completely reaches swelling equilibrium at 20 h. The mass change is 52.3% soaked in water and 67.9% soaked in saline (Fig. S10b). Notably, the prepared saline contains 26.7 g L^−1^ of NaCl and 7 g L^−1^ of KCl, which are the main salt components of seawater (56). NaCl and KCl have salting-in attributes for the hydrogel network according to the Hofmeister effect (57,58), consequently the hydrogel presents higher swelling properties in saline. In addition, the salt ions in the hydrogel could leak when soaked in water and saline, resulting in mass exchange. Therefore, we further explored the volume swelling of the PAA/Cel hydrogel by soaking in water and saline. As shown in Fig. S10c, after the PAA/Cel hydrogel is stabilized in water and saline for 20 h, the hydrogel shows volume swelling to some extent, but still maintains macroscopic integrity without rupture due to swelling. The PAA/Cel hydrogel exhibits a similarly higher volume change of 307.7% in saline compared to 222.3% in water (Fig. S10d).


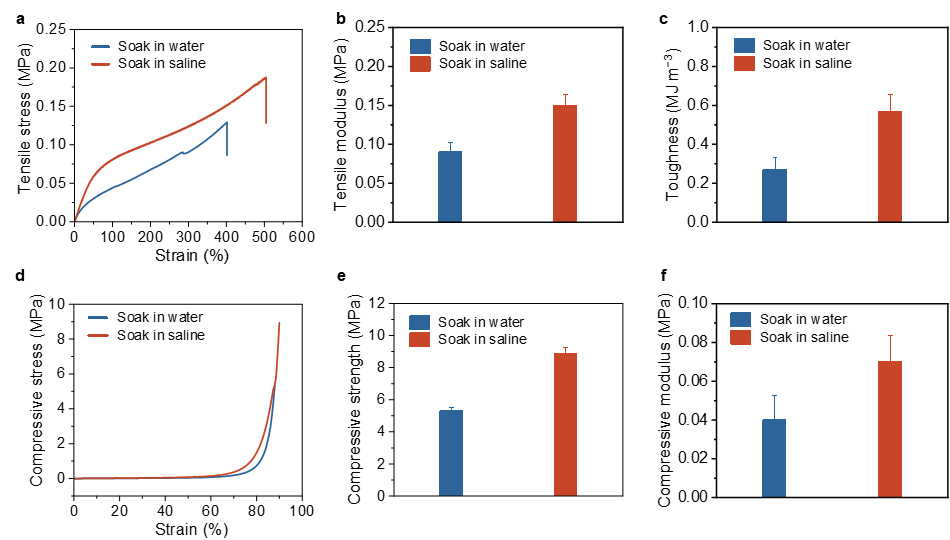


**Figure. S11 Mechanical preparties of the PAA/Cel hydrogel in water and saline. a−c** Tensile stress-strain curves (**a**), tensile modulus (**b**), and toughness (**c**) of the PAA/Cel hydrogel after stabilization in water and saline. **d−f** Compressive stress-strain curves (**d**), compressive strength (**e**), and compressive modulus (**f**) of the PAA/Cel hydrogel after stabilization in water and saline.

The tensile stress-strain curve of the PAA/Cel hydrogel is shown in Fig. S11a. After the hydrogel is soaked in water to reach equilibrium, it shows an ultimate stress of 0.13 MPa at a fracture strain of 401.5%, corresponding to that of 0.19 MPa at a fracture strain of 504.7% in saline. The tensile modulus and toughness of the PAA/Cel hydrogel in saline are 1.7 times (0.15 MPa vs. 0.09 MPa) and 2.1 times (0.57 MJ m^−3^ vs. 0.27 MJ m^−3^) higher than those in water respectively (Fig. S11b and c). In cases of compression properties, the PAA/Cel hydrogel shows similar mechanical behavior (Fig. S11d). Typically, compared with in water, the PAA/Cel hydrogel in saline exhibits 1.7 times (8.9 MPa vs. 5.3 MPa) higher in compressive strength, and 1.8 times (0.07 MPa vs. 0.04 MPa) higher in compressive modulus (Fig. S11e and f). Therefore, these favorable mechanical observations demonstrate that the PAA/Cel hydrogel have excellent mechanical properties both in water and saline, especially in saline.


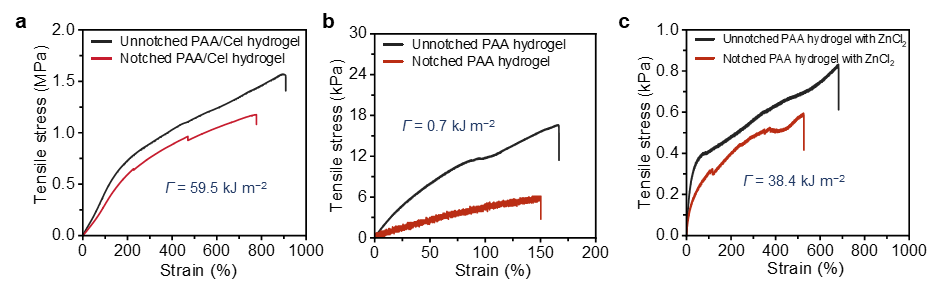


**Figure. S12 a** Tensile stress-strain curves of notched and unnotched PAA/Cel hydrogels. **b** Tensile stress-strain curves of notched and unnotched PAA hydrogels. **c** Tensile stress-strain curves of notched and unnotched PAA hydrogel with ZnCl_2_.


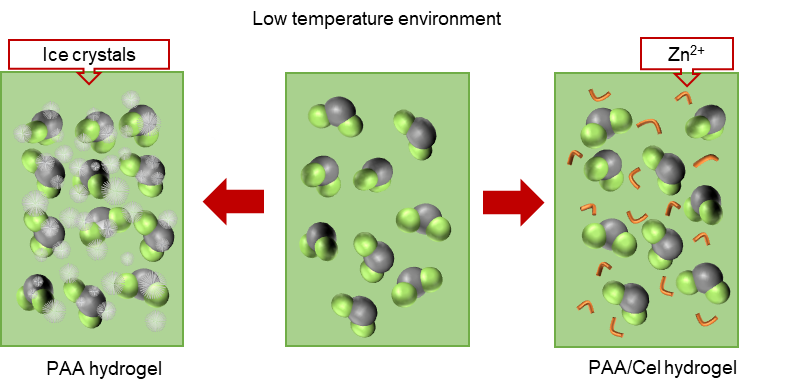


**Figure. S13** Schematic illustration of the mechanism of cryogenically adaptive properties of the PAA/Cel hydrogel.


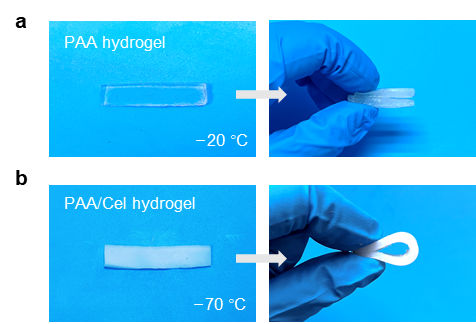


**Figure. S14** **a** Photographs of the PAA hydrogel fractured at −20 °C showing brittleness due to freezing. **b** Photographs of the PAA/Cel hydrogel that can be bent at −70 °C showing flexibility.


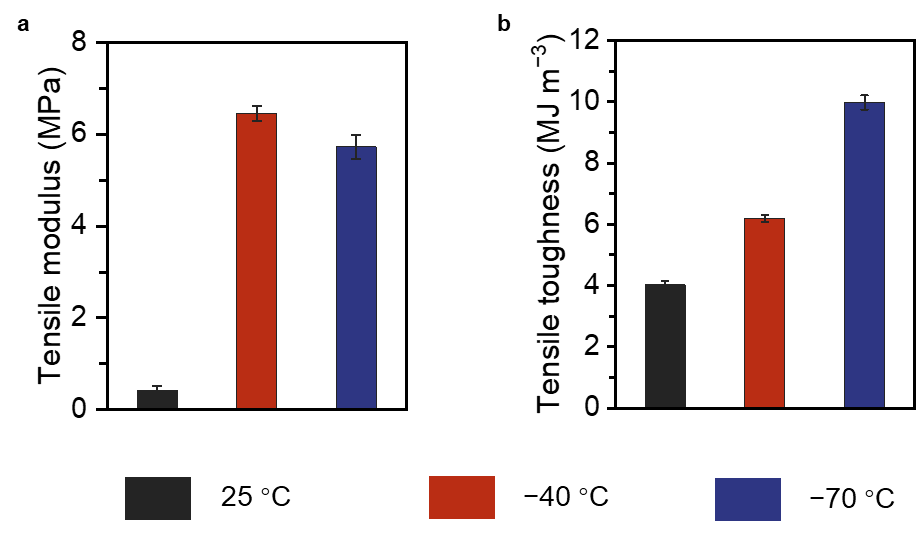


**Figure. S15** **a, b** A comparison of tensile modulus (**a**) and tensile toughness (**b**) of the PAA/Cel hydrogel at 25, −40, and −70 °C, respectively.


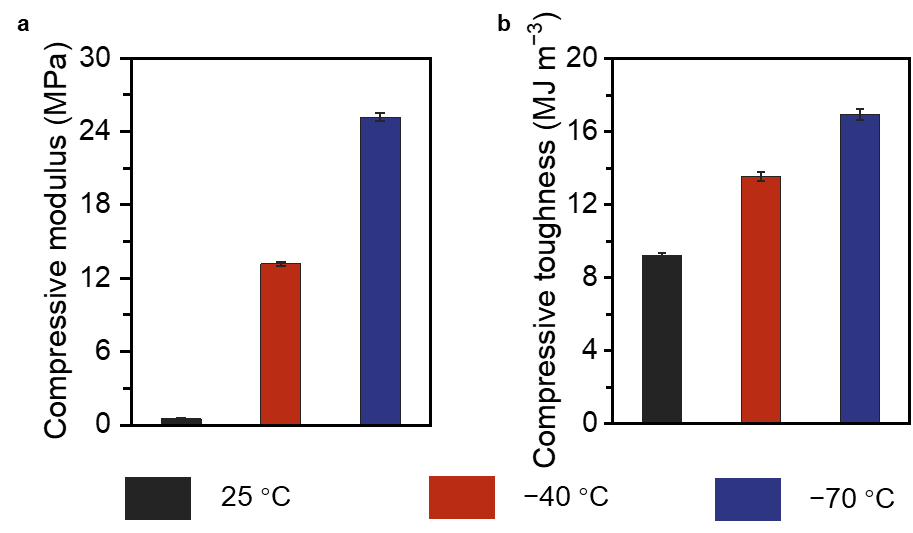


**Figure. S16** **a, b** A comparison of compressive modulus (**a**) and compressive toughness (**b**) of the PAA/Cel hydrogel at 25, −40, and −70 °C, respectively.


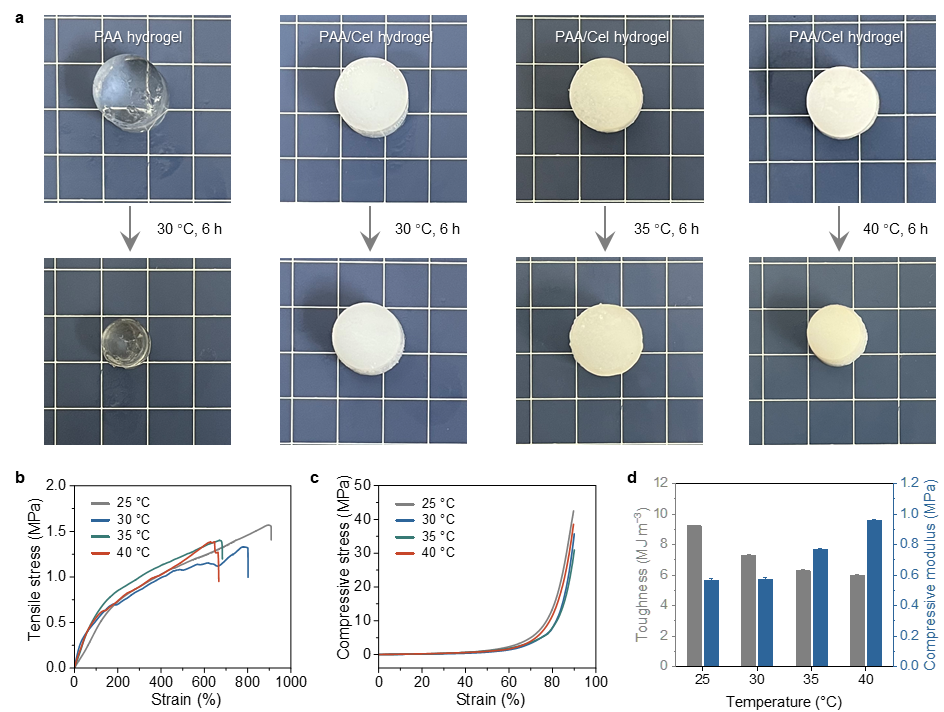


**Figure. S17 a** Photographs of the PAA hydrogel and PAA/Cel hydrogel before and after stabilization at varying temperatures. **b, c** Tensile (**a**) and compressive (**c**) stress-strain curves of the PAA/Cel hydrogel at 25, 30, 35 and 40 °C. **d** Toughness and compressive modulus of the PAA/Cel hydrogel at 25, 30, 35 and 40 °C.

As shown in Fig. S17a, the PAA hydrogel exhibits intense shrinkage behavior when stored at 30 °C for 6 h due to the evaporation of a large amount of water causing aggregation of the polymer backbone in the hydrogel network. For the PAA/Cel hydrogel, a negligible shrinkage is observed after 6 h at 30 and 35 °C to reach equilibrium; however, a visible shrinkage is also observed at 40 °C, demonstrating its excellent stability when exposed to temperatures below 35 °C. The tensile and compressive properties of the PAA/Cel hydrogel were further quantitatively studied at varying temperatures (Fig. S17b and c). We can observe that as the temperature increases from 25 °C to 40 °C, the hydrogels exhibit decreased toughness (9.2 to 5.9 MJ m^−3^) and increased compressive modulus (0.6 to 1.0 MPa) (Fig. S17d). This is because the increase in temperature leads to the polymer to aggregate to a certain extent, which results in a more rigid hydrogel. However, this is still acceptable for practical use because the stretchability of the PAA/Cel hydrogel remains above 630%.


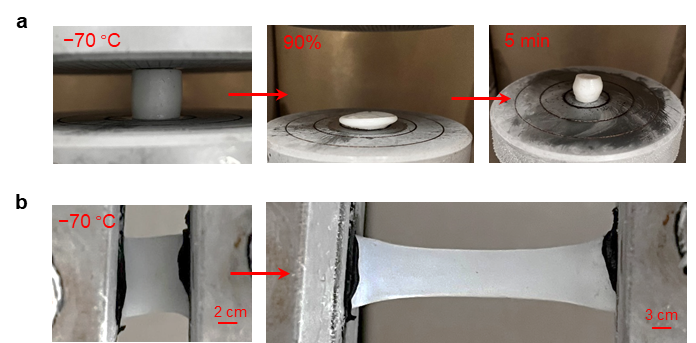


**Figure. S18** **a, b** Photographs of the PAA/Cel hydrogel subjected to compressive (**a**) and tensile (**b**) tests at −70 °C.

**Figure. S19** Corresponding stress of the PAA/Cel hydrogel at maximum compressive strain of 50% at 25 °C and −70 °C.

**Figure. S20** EIS plots of the PAA/Cel hydrogel at 25, −10, −30, −50, and −70 °C, respectively.


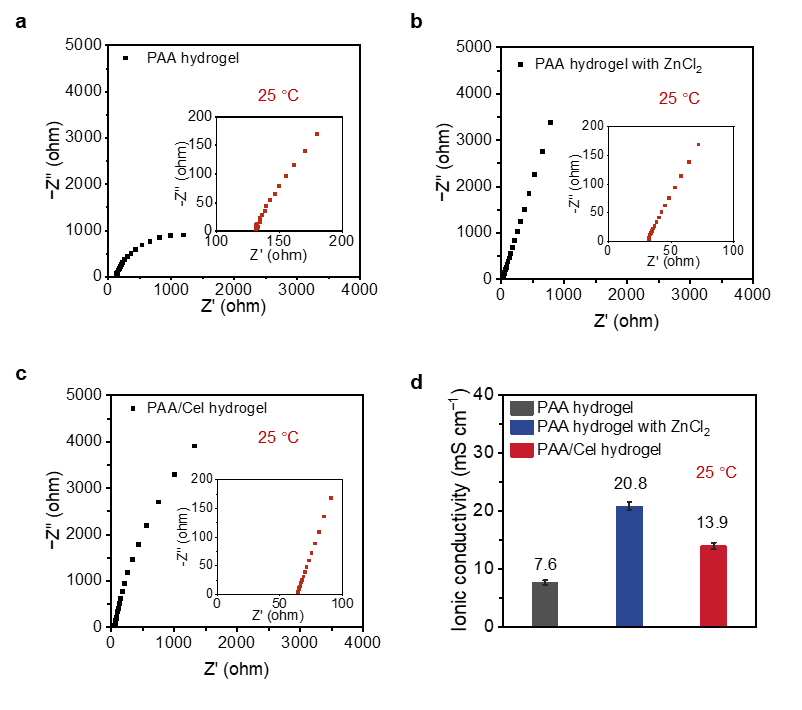


**Figure. S21** **a−c** EIS plots of the PAA hydrogel (**a**), PAA hydrogel with ZnCl_2_ (**b**), and PAA/Cel hydrogel (**c**) at 25 °C. **d** A comparison of ionic conductivity of the PAA hydrogel, PAA hydrogel with ZnCl_2_, and PAA/Cel hydrogel at 25 °C.


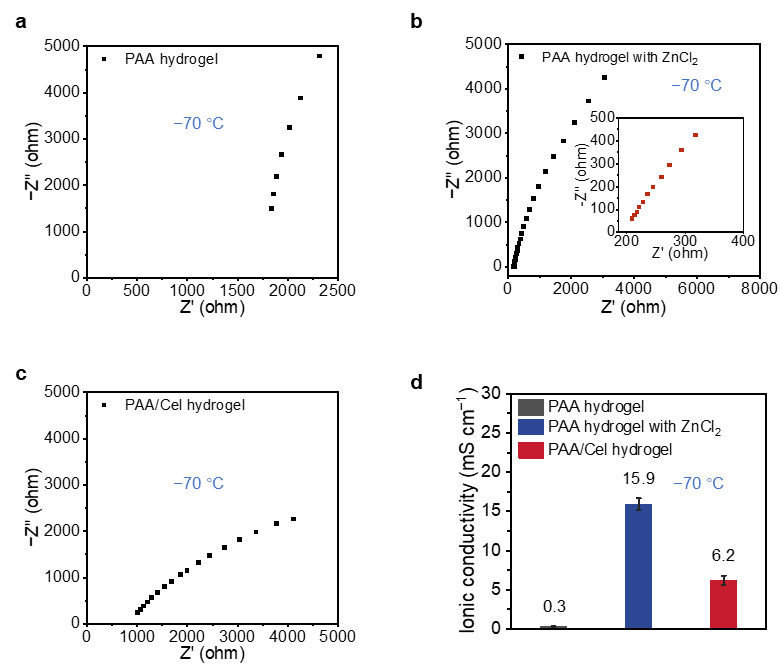


**Figure. S22** **a−c** EIS plots of the PAA hydrogel (**a**), PAA hydrogel with ZnCl_2_ (**b**), and PAA/Cel hydrogel (**c**) at −70 °C. **d** A comparison of ionic conductivity of the PAA hydrogel, PAA hydrogel with ZnCl_2_, and PAA/Cel hydrogel at −70 °C.


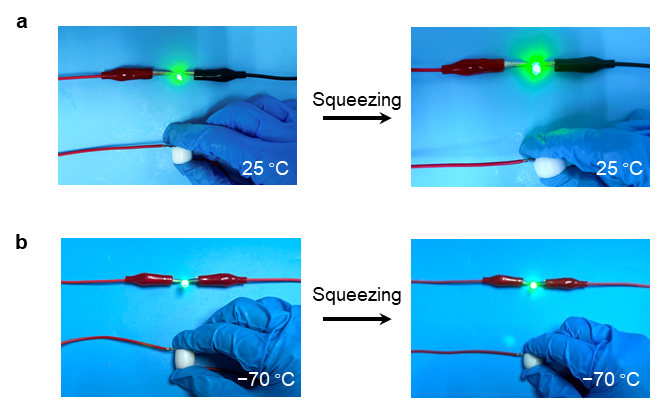


**Figure. S23** **a, b** Photographs of the PAA/Cel hydrogel ionic conductor forming a closed loop before and after being squeezed to light up LED lights at 25 (**a**) and −70 °C (**b**), respectively.


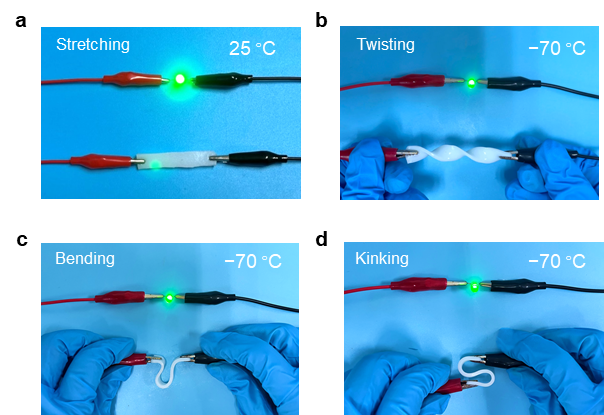


**Figure. S24** **a** Photographs of the PAA/Cel hydrogel ionic conductor forming a closed loop after stretching to light up LED lights at 25 °C. **b−d** Photographs of the PAA/Cel hydrogel ionic conductor forming a closed loop after twisting (**b**), bending (**c**), and kinking (**d**) to light up LED lights at −70 °C, respectively.

**Figure. S25** Continuous resistance changes of the PAA/Cel hydrogel ionic conductor as e−skins at compressive strains of 0%, 25%, 50%, and 75% for 20 s, respectively.


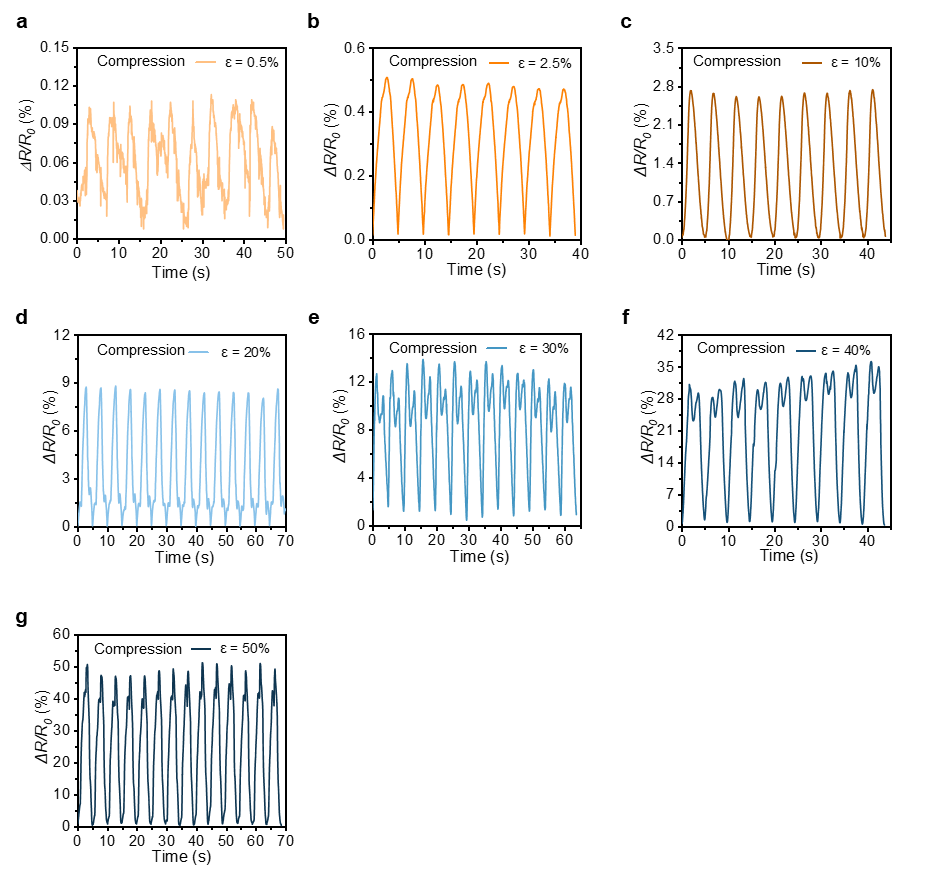


**Figure. S26 a−g** Continuous resistance changes of the PAA/Cel hydrogel ionic conductor as e−skins at compressive strains of 0.5% (**a**), 2.5% (**b**), 10% (**c**), 20% (**d**), 30% (**e**), 40% (**f**), 50% (**g**), respectively.

**Figure. S27** Real-time responses by compression the PAA/Cel hydrogel ionic conductor to 50% strain for 1000 cycles at 25 and −70 °C, respectively.


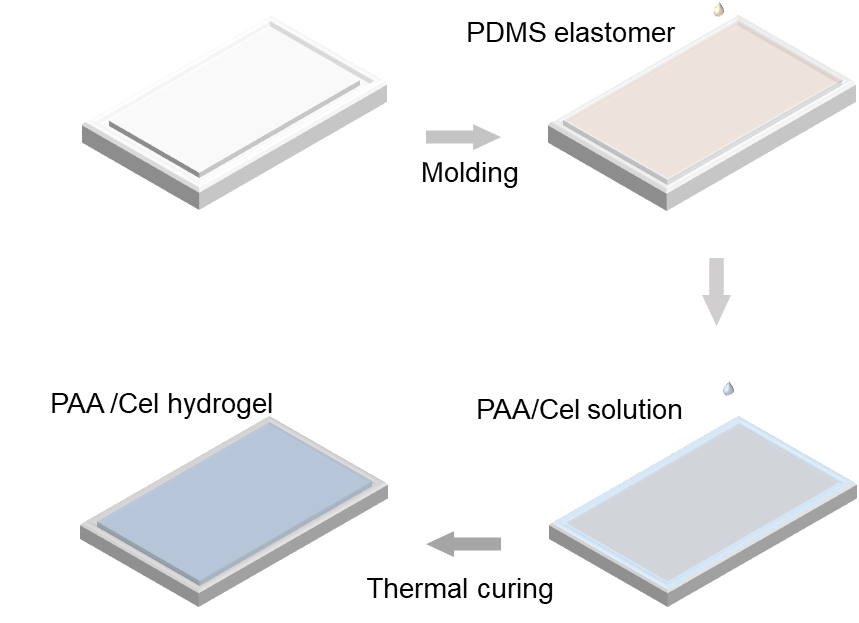


**Figure. S28** Schematic illustration of fabricating the two-layer structured hydrogel-based TENG.


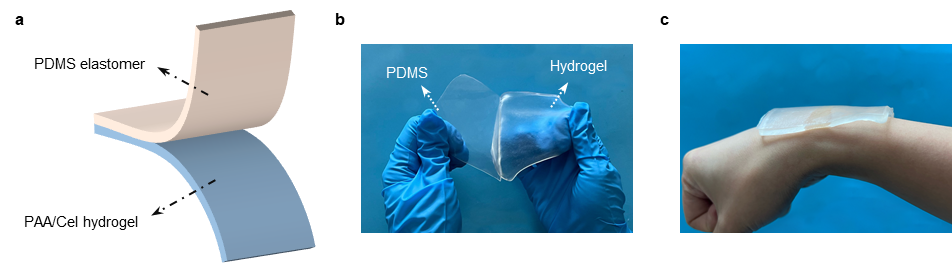


**Figure. S29** **a** Hydrogel-based TENG was composed of a silicone rubber layer (PDMS) as the tribo-negative material and the PAA/Cel hydrogel as both the ionic current collector. **b** Photograph of the hydrogel and PDMS being pulled under external force. **c** Photograph of the hydrogel-PDMS integrated material fixed at the wrist after continuous bending.

As shown in Fig. S29a, the resulting PAA/Cel hydrogel and PDMS could be assembled together to form a hydrogel-elastomer integrated material. We found that it is difficult to separate the hydrogel and PDMS even when they are pulled under the action of external force (Fig. S29b). When the obtained hydrogel-elastomer integrated material is fixed on the wrist and subjected to continuous bending, the hydrogel and elastomer still maintains a tight fit without significant separation gaps, proving its great practical applicability (Fig. S29c).


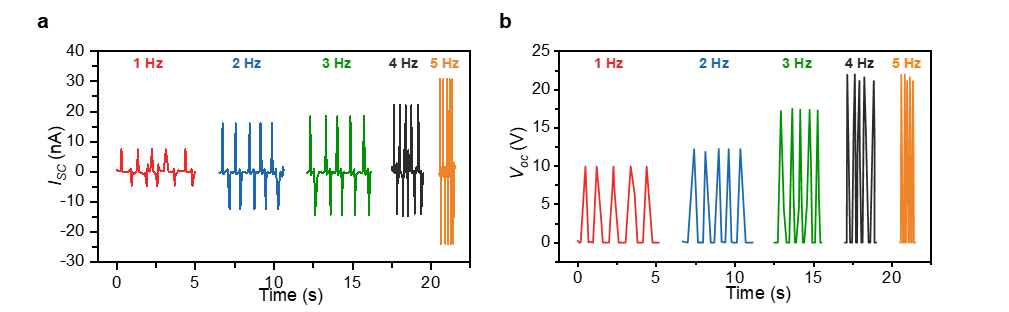


**Figure. S30** **a, b** Short-circuit current (**a**) and open-circuit voltage (**b**) curves of the PAA/Cel hydrogel-based TENG in the frequency range of 1–5 Hz.

**Table S1** Summary of comprehensive properties of hydrogels.

| References | Types | Ionic conductivity  (mS cm^−1^) | Freezing temperature (°C) | Compressive strength (MPa) | Tensile strength (MPa) | Toughness  (MJ m^−3^) | Fracture strain (%) |
| --- | --- | --- | --- | --- | --- | --- | --- |
| This work | PAA/Cel hydrogel | 13.9 | N/A | 42.5 | 1.6 | 9.2 | 896.9 |
| Ref (32) | PAM/CS hydrogel | 10.8 | −45 | 17.3 | 0.9 | 1.9 | 320 |
| Ref (29) | P(SBMA-co-HEA) hydrogel | 12.6 | −44.5 | 0.04 | 0.005 | 0.7 | 325 |
| Ref (30) | PVA/PEDOT:PSS hydrogel | 0.09 | −40 | 1.2 | 2.1 | 6.8 | 715 |
| Ref (22) | PVA/CNF/DMSO hydrogel | 3.2 | −70 | 16.3 | 2.1 | 5.2 | 660 |
| Ref (31) | PAA/gelatin/TA hydrogel | 3.1 | −37 | 9.4 | 0.34 | 2.87 | 900 |

**Table S2** Summary of tensile and compressive properties of hydrogels.

| References | Types | Compressive strain (%) | Compressive strength (MPa) | Tensile strain (%) | Tensile strength (MPa) |
| --- | --- | --- | --- | --- | --- |
| This work | PAA/Cel hydrogel | 90 | 42.5 | 896.9 | 1.6 |
| Ref (47) | CS/PACG-Fe^3+^  DN hydrogel | 97 | 36.8 | 240 | 1.2 |
| Ref (49) | Silk fibroin/gelatin hydrogel | 75 | 0.8 | 200 | 0.1 |
| Ref (22) | PVA/CNF/DMSO hydrogel | 90 | 16.7 | 363 | 2.1 |
| Ref (32) | PAM/CS hydrogel | 95 | 17.6 | 320 | 0.9 |
| Ref (46) | PVA/P(iAA) hydrogel | 95 | 12.4 | 614 | 1.5 |
| Ref (48) | P(AM−APBA) hydrogel | 90 | 1.4 | 810 | 0.2 |

**Table S3** Summary of fracture strain of hydrogels at low temperatures.

| References | Types | Tensile temperature (°C) | Fracture strain (%) |
| --- | --- | --- | --- |
| This work | PAA/Cel hydrogel | −70 | 595 |
| This work | PAA/Cel hydrogel | −40 | 730 |
| Ref (17) | PVA/DMSO hydrogel | −30 | ~200 |
| Ref (43) | PAA/betaine hydrogel | −40 | ~300 |
| Ref (32) | PAM/CS hydrogel | −30 | 320 |
| Ref (51) | PVA/LiCl hydrogel | −40 | 330 |
| Ref (52) | PAM/PEG hydrogel | −20 | 650 |

**Table S4** Summary of properties of hydrogels presented in this work.

| Parameters | PAA/Cel hydrogel | PAA hydrogel with ZnCl_2_ | PAA hydrogel |
| --- | --- | --- | --- |
| Tensile stain (%) | 896.9 | 682.5 | 166.1 |
| Tensile strength (MPa) | 1.6 | 0.8 | 0.02 |
| True tensile strength (MPa) | 14.1 | 5.7 | 0.03 |
| Compressive strength (MPa) | 42.5 | 17.5 | 11.9 |
| Young’s modulus (MPa) | 0.4 | 1.7 | 0.02 |
| Toughness (MJ m^−3^) | 9.2 | 3.9 | 0.02 |
| Notched tensile strain (%) | 776.2 | 526.7 | 150.3 |
| Notched tensile strength (MPa) | 1.2 | 0.6 | 0.006 |
| Ionic conductivity at 25 °C  (mS cm^−1^) | 13.9 | 20.8 | 7.6 |
| Ionic conductivity at −70 °C  (mS cm^−1^) | 6.2 | 15.9 | 0.3 |
| Freezing temperature (°C) | N/A | N/A | −18.6 |
| Fracture energy (kJ m^−2^) | 59.5 | 38.4 | 0.7 |

**References**

56. Ren J, Liu Y, Wang Z, Chen S, Ma Y, Wei H*, et al.* An anti‐swellable hydrogel strain sensor for underwater motion detection. *Adv Funct Mater* 2021, **32**(13)**:** 2107404.

57. Hua M, Wu S, Ma Y, Zhao Y, Chen Z, Frenkel I*, et al.* Strong tough hydrogels via the synergy of freeze-casting and salting out. *Nature* 2021, **590**(7847)**:** 594-599.

58. Wu S, Hua M, Alsaid Y, Du Y, Ma Y, Zhao Y*, et al.* Poly(vinyl alcohol) hydrogels with broad-range tunable mechanical properties via the Hofmeister effect. *Adv Mater* 2021, **33**(11)**:** e2007829.
